# A Rare Case of Systemic Lupus Erythematosus With Diffuse Alveolar Hemorrhage and Guillain-Barre Syndrome

**DOI:** 10.7759/cureus.33984

**Published:** 2023-01-20

**Authors:** Zaher A Althagafi, Shahad S Al-Bishi, Riyazuddin Ansari, Hassan A Alsolami, Lamyaa G Abdelkader

**Affiliations:** 1 Rheumatology, King Faisal Hospital, Makkah, SAU; 2 Critical Care, King Faisal Hospital, Makkah, SAU; 3 Gastroenterology, King Faisal Hospital, Makkah, SAU

**Keywords:** steroids, cyclophosphamide, diffuse alveolar hemorrhage, gbs, systemic lupus erythematosus

## Abstract

Systemic lupus erythematosus (SLE) is an autoimmune systemic disease with many organ involvements with high morbidity and mortality percentage. It's unusual for systemic lupus erythematosus (SLE) to present with diffuse alveolar hemorrhage (DAH) as the earliest presentation. Diffuse alveolar hemorrhage (DAH) refers to the effusion of blood into the alveoli due to damaged pulmonary microvasculature. It’s a rare but severe complication of systemic lupus associated with a high mortality rate. It occurs in three different overlapping phenotypes, which are acute capillaritis, bland pulmonary hemorrhage, and diffuse alveolar damage. diffuse alveolar hemorrhage develops in a short period of time (hours to days). Central and peripheral nervous system complications generally develop during the course of the illness and actually uncommonly from the beginning of the illness. Guillain-Barre syndrome (GBS) is a rare autoimmune polyneuropathy usually occurring post-viral, post-vaccination, or surgery. Systemic lupus erythematosus (SLE) has been associated with several neuropsychiatric manifestations and the development of GBS. GBS as the first presentation of SLE is exceedingly rare. Here, we present the case of a patient with diffuse alveolar hemorrhage and Guillain-Barre syndrome as an atypical presentation of systemic lupus erythematosus (SLE) flare.

## Introduction

Systemic lupus erythematosus (SLE) is a multisystemic, chronic, inflammatory, recurrent, autoimmune illness with various clinical manifestations. One of the most harmful side effects of systemic lupus erythematosus is diffuse alveolar hemorrhage, which was initially identified in 1904 by Osler [1]. Although mortality rates have decreased in recent generations for diffuse alveolar hemorrhage (DAH), reported rates are still between 0% and 92% (with an average of 50%). Diffuse alveolar hemorrhage can present with acute hemoptysis, renal failure, and respiratory failure leading to the necessity for mechanical ventilation, sepsis, and thrombocytopenia and are all related to a higher risk of death [2].

In SLE, neurological problems are prevalent and common. Involvement of the central nervous system (CNS) is one of the most frequent side effects that can develop at any stage of SLE. Although peripheral nervous system involvement in SLE is rare, it manifests as mononeuropathies multiplex and distal symmetric axonal polyneuropathy [3]. It is extremely uncommon to have either the classic form of Guillain-Barre syndrome (GBS) or acute inflammatory demyelinating polyneuropathy (AIDP). In our case, we provide severe diffuse alveolar hemorrhage and Guillain-Barre syndrome as the presenting manifestation of SLE in a 20-year-old female who developed severe hypoxemia leading to mechanical ventilation.

## Case presentation

A 20-year-old female with no past medical history, presented to our emergency department complaining of fever, vomiting 10 times (coffee ground) four attacks of diarrhea (green in color, no melena). History of dysuria with lower abdominal pain, headache, ear pain, oral ulcer (lower lip), generalized weakness, and one episode of epistaxis.

**Figure 1:**
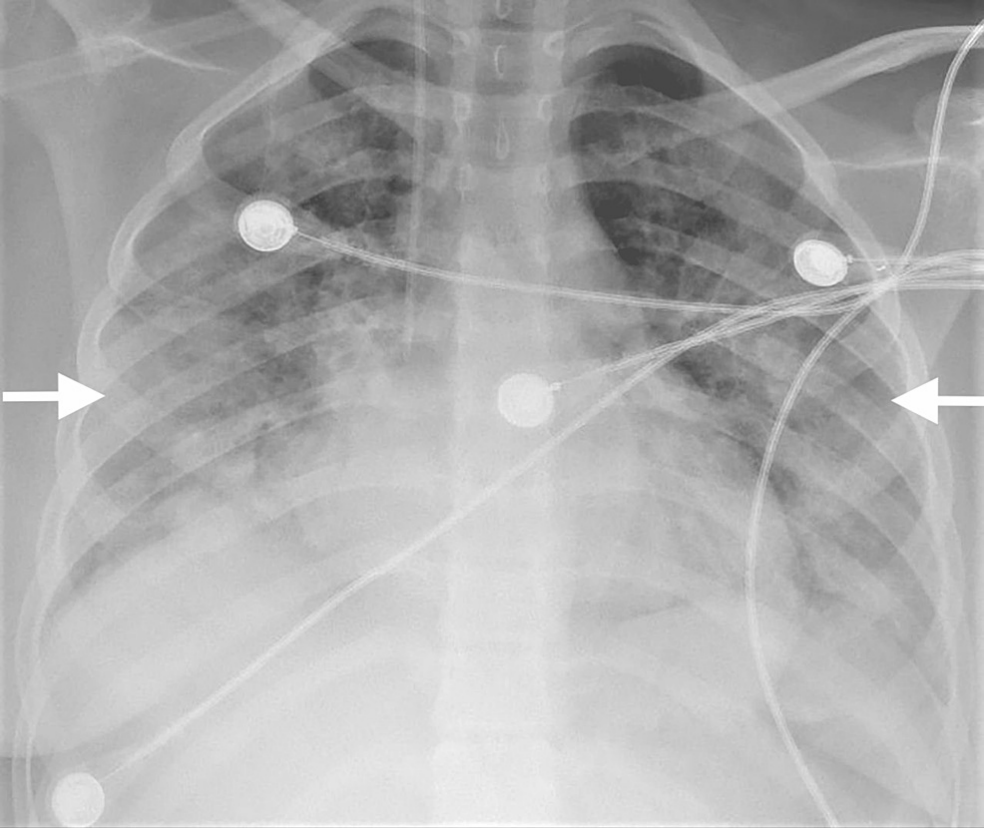
Chest x-ray showing bilateral pulmonary infiltrates.

On presentation, the patient arrived at intensive critical care hypoxic on high-flow oxygen. She was hypotensive and immediately intubated. Was started on low dose of noradrenaline infusion. Chest x-ray showed bilateral pulmonary infiltrates (Figure 1).

Laboratory investigation revealed hemoglobin 6.39 g, platelet 11,400,00, and WBC 15,800. Anti-nuclear antibodies (ANA) and anti-dsDNA were positive with 1:1280 titer (fluorescence pattern, homogenous). Twenty-four hours urine protein showed a total protein of 17.778 g/day. Urine creatinine was 13,900 μmol/L. C3 level was <40 mg/dL and C4 <8 mg/dL. C-reactive protein was 96 mg/dL with all cultures negative.

**Figure 2:**
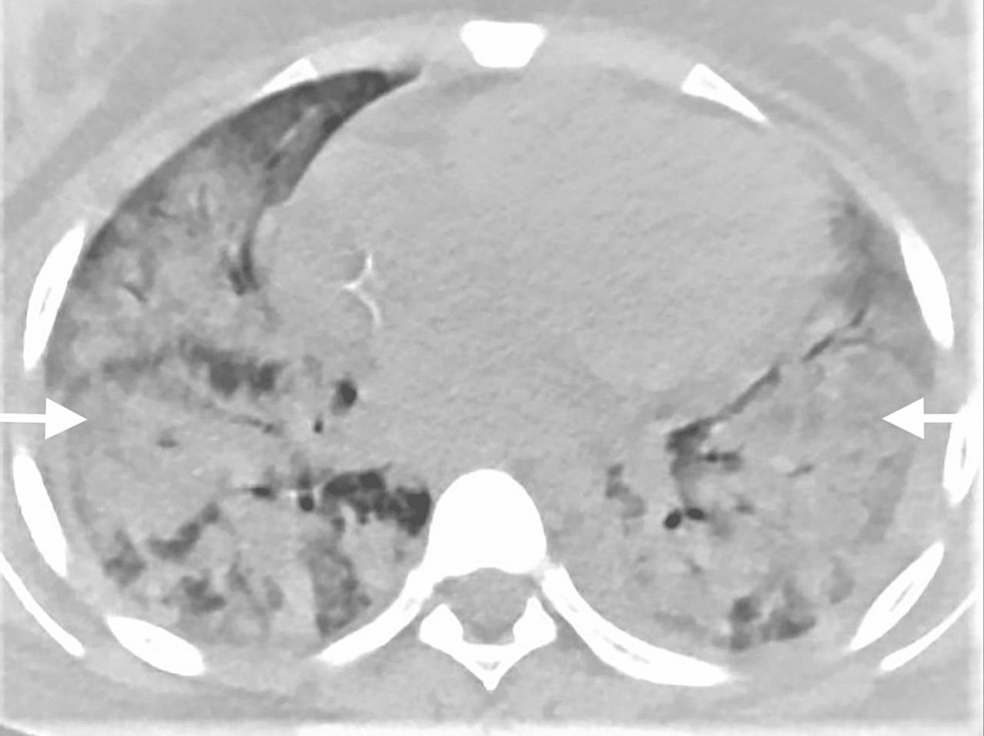
High-HRCT before treatment showing bilateral diffuse alveolar hemorrhagic infiltration. HRCT: resolution computed tomography

Echocardiography showed a left ventricular ejection fraction of around 52%, septal hypokinesia with mild tricuspid regurgitation, and right ventricular systolic pressure of 24 mmHg. The patient underwent esophagogastroduodenoscopy which revealed hyperemic gastric mucosa, more in the fundus with a hemorrhagic spot. No features of vasculitis or *Helicobacter pylori *organisms were seen in the submitted biopsy. After three days of intubation, the patient was extubated and was conscious and oriented. After the patient became stable, she underwent high-resolution computed tomography (HRCT) which showed bilateral diffuse alveolar hemorrhagic infiltration (Figure 2).

We performed chest computerized tomography with contrast which didn’t reveal any radiological evidence of pulmonary embolism. Hence, we repeated the echocardiograph which revealed tachycardia, ejection fraction of 53% with septal mid-hypokinesia, and mild mitral regurgitation suspected of lupus myocarditis. Ultrasound abdomen was normal. We couldn’t perform bronchoscopy since her oxygen requirement was around 15 L/min. We decided to start pulse methylprednisolone 1 g daily for five days. Since the response was weak, the decision of plasma exchange six sessions every other day, intravenous immunoglobulin (IVIG) 0.4 g/kg/day with total of five doses with intravenous cyclophosphamide 500 mg every two weeks for six doses, and extended pulse therapy for seven days was taken. After one session of plasma exchange, the further sessions were stopped due to the effect of plasma exchange on cyclophosphamide. The patient had lower limb weakness and a nerve conduction study was performed which revealed marked reduced motor amp of all tested nerves, positive temporal dispersion, and absent F wave of right ulnar, preserved sensory nerves with evidence of acute motor axonal neuropathy (AMAN) type of Guillain-Barre syndrome (GBS). The treatment plan was revised to cyclophosphamide Euro-Lupus 500 mg every two weeks for six doses, intravenous immunoglobulin (IVIG) for five days, and trimethoprim + sulfamethoxazole 960 mg orally three times weekly. The patient after 15 days in ICU showed dramatic improvement and was shifted to the ward conscious, and oriented. She was vitally stable on 2 L oxygen nasal cannula. In the ward, second high-resolution computed tomography (HRCT) was performed on the patient to rule out the progression of alveolar hemorrhage and it showed an interval decrease in the amount and shape of previous alveolar hemorrhage.

She was discharged from the hospital on room air with no weakness. She continued to follow up with nephrology and rheumatology OPD to complete the last two doses of cyclophosphamide in daycare unit. Her last laboratory investigation showed urine protein/creatinine of 176 μmol/L with urine creatinine of 1015 μmol/L. The urine protein random was 179 mg/L. We also repeated C3 level which improved to 104.6 mg/dL with c-reactive protein 0.16 mg/dL. She will continue to receive cyclophosphamide (one dose remaining).

## Discussion

Systemic lupus erythematosus (SLE) rarely develops the complication of diffuse alveolar hemorrhage (DAH). it's rare as the initial manifestation of lupus [4]. DAH in SLE is considered life-threatening with mortality rates of about 50% [4]. DAH pathophysiology involves pulmonary capillaritis, soft hemorrhages, and immune complexes deposition on the alveolar wall which damages the basement membranes and allows erythrocytes to escape into the alveolar space [2]. Lupus nephritis typically occurs within three to five years of the onset of SLE. Nephritis (approximately 70% of cases) is the organ involvement most frequently associated with SLE cases with DAH. Lupus nephritis is primarily due to type 3 hypersensitivity reaction which causes the formation of immune complexes. This together with anti-dsDNA binds to the mesangium. This results in an inflammatory response leading to lupus nephritis. Anti-dsDNA was high in 75% of cases and low complement in 86% of cases [5]. To effectively treat patients of SLE associated with DAH, more randomized clinical trials are needed, and management is unified across all health institutions [6]. Plasmapheresis, cyclophosphamide, and methylprednisolone are the treatments that are most frequently used [7]. Corticosteroids were the most frequently used (98%) in one study involving 140 cases followed by cyclophosphamide (54%), plasmapheresis (31%), azathioprine (7%), intravenous immunoglobulin (IVIG, 5%), mycophenolate (3%), the B cell targeted therapy rituximab (RTX, 6%), and stem cell transplantation (2%) [5]. The combination of methylprednisolone and cyclophosphamide which is used in various centers has been linked to a higher rate of survival [8]. Patients who were treated with cyclophosphamide showed a better response and prolonged life span as compared to plasmapheresis [5].

In 10-20% of SLE cases, the peripheral nervous system presents with polyneuropathy [9]. Nonetheless, GBS is a demyelinating polyneuropathy and an uncommon complication of lupus [10]. SLE with GBS occurs between 0.6% and 1.7%, which indicates its rarity [11]. The first case of SLE with GBS manifestation was reported in 1964 [12]. The diagnosis of Guillain-Barre syndrome in our case was predicated on limb symmetry, flaccid paralysis, and electromyography demonstrating demyelinating polyneuropathy AMAN type. Our case met four criteria of the American College of Rheumatology for SLE which were renal involvement (nephritis), anemia, positive ANA, and positive ds-DNA antibodies.

The pathogenesis of GBS in SLE involves vascular occlusion of small vessels due to vascular endothelial hyperplasia. Many anti-neuronal antibodies like anti-cardiolipin antibodies, anti-lymphocytic antibodies, and lupus anti-coagulants cause damage to the myelin components of the nerves [13].

Neuropsychiatric systemic lupus erythematosus (NPSLE) which is usually due to an inflammatory pathway causing immune complex formation and breaking the blood-brain barrier is fatal in SLE patients [14]. Literature has recommended various modalities of treatment for GBS with SLE, like cyclophosphamide, corticosteroids, plasmapheresis, and immunoglobulin. Solitary corticosteroid treatment didn’t have a good outcome [15].

Regarding the treatment, we treated our patient with pulse therapy methylprednisolone 1000 mg IV once daily for seven days, cyclophosphamide Euro-Lupus 500 mg every two weeks for six doses, intravenous immune globulin (IVIG) 0.4 g/kg per day for a total of five days and trimethoprim + sulfamethoxazole 960 mg orally three times weekly. The patient is still following up with the rheumatology OPD to complete the cyclophosphamide course. During the follow-up, she is conscious, oriented, and fully mobile without any aids.

## Conclusions

DAH and GBS are unexpected presenting manifestations and potentially fatal complications of SLE, we need to be vigilant about the development of GBS and DAH which if left untreated can lead to high mortality. Early and aggressive therapy should be implemented in such kind of patients with the use of steroids, cyclophosphamide, and IVIG.
